# Supplementary material for: Are carbon emissions trading and green financial instruments synergistic? -Comprehensive quantitative research based on content analysis
Source: PLoS One. 2024 Mar 7;19(3):e0298601. doi: 10.1371/journal.pone.0298601 (PMC10919601; doi:10.1371/journal.pone.0298601)
Supplement: S1 Data — (ZIP) [file pone.0298601.s001.zip › Data/地级市Z_F工作报告.zip!/║┌┴·╜¡/┤≤╟∞╩╨/└┤╫╘╣1⁄2╓┌║┼í╛┬φ┐╦╩2╛▌═°í┐_2011.docx]

各位代表：

　　现在，我代表市人民政府向大会作工作报告，请予审议，并请市政协委员和列席会议的同志提出意见。

　　一、2010年工作回顾和“十一五”发展成就

　　过去的一年，我们按照市委“全党抓发展，中心抓经济，重点抓项目”的总体要求，深入贯彻“一二三 四五”发展战略，着力打牢“四个基础”，突出优化经济结构和转变发展方式，大力提升城市功能和改善生态环境，努力增强县域经济实力和缩小城乡差距，着力改善人民生活和维护社会稳定，全面完成了市八届人大五次会议确定的目标任务。全市实现地区生产总值2900亿元、增长12%，全社会固定资产投资1036亿元、增长37.5%，地方财政收入175.5亿元、增长20.6%，城镇居民人均可支配收入和农民人均纯收入分别突破20000元和8000元、增长10.1%和22%，万元gdp综合能耗下降4.1%。

　　（一）油化支撑、地方壮大，工业经济持续快速增长。实现工业增加值2320亿元、增长11.3%。原油保持稳产。生产原油4000万吨、天然气30亿立方米，实现增加值1500亿元、增长1.8%。石化扩能增效。120万吨柴油加氢、5.2万吨聚丙烯酰胺等项目建成投产，120万吨乙烯改扩建、30万吨聚丙烯二期等大项目加快推进，规模以上石化工业实现增加值360亿元、增长27.8%。地方工业高速增长。建设投资千万元以上项目245个，其中亿元以上项目72个。大庆联谊石化恢复生产、实现销售收入20亿元，展华生化科技等企业加快壮大，全市净增规模以上企业181家，规模以上地方工业实现增加值290亿元、增长55%。园区经济加快发展。高新区实现工业增加值261亿元、增长56%，倍增目标如期实现。经开区、林源工业区以及各县区专业园区功能整体提升，全市园区工业增加值增长55%。

　　（二）农业增效、面貌改观，新农村建设成效明显。实现农业增加值95亿元、增长10%。“双十”工程扎实推进。农业综合发展能力增强，新增膜下滴灌93万亩、旱涝保收田62.4万亩、土地改良1.5万亩，发展农机合作社46个、农村经济合作组织71个，推广农业先进技术155项，培训农民36万人次。农村面貌发生较大变化，新建农村公路368公里、饮水安全工程256处、沼气入户工程50项，新增上网村50个，有线电视入户3.4万户，实施农村电网升级改造工程26项，整体绿化村屯346个，造林23.1万亩。农民保障水平进一步提高，医保人均筹资标准和大病补助标准分别达到150元以上和3万元以上，1.3万困难农户实现脱贫。“四大基地”建设取得突破。粮食产量达到101.1亿斤，首次突破百亿斤大关；奶牛存栏达到48.5万头，肉牛和生猪饲养量分别达到49.4万头和310万头，畜牧业增加值增长10%；绿色食品基地发展到450万亩，“三品”标识达到380个；启动建设四季青温泉果蔬基地、八井子现代农业主题观光园、红骥牧场农业特色风情园等设施农业项目，全市棚室发展到4.8万栋，设施农业实现收入25亿元。（三）业态升级、市场繁荣，服务业保持良好发展态势。实现服务业增加值420亿元、增长16.3%。商贸业层次提升。重点建设万达广场等项目15个，苏宁电器等知名企业落户大庆，促进消费持续旺盛，社会消费品零售总额实现591亿元、增长23.8%。旅游业影响扩大。启动建设北国之春梦幻城、龙凤国家城市湿地公园等29个项目，连环湖温泉景区一期、北国温泉养生广场等建成投用，成功举办第三届湿地旅游文化节，实现旅游收入26亿元、增长33%。文化产业实现突破。新华08国际石油资讯中心开工建设，百湖影视基地等项目加快推进，“黎明河休闲水岸”建成开业，休闲娱乐消费成为新热点，文化产业实现营业收入65亿元、增长26.2%。服务外包实力增强。新增企业61家，全市服务外包业务执行额实现67亿元，离岸外包业务在全国21个服务外包示范城市中位列第10位。物流业加快发展。铁路西货站竣工投用，神舟石化物流园投入运营，新站粮食物流中心等建设扎实推进，物流企业发展到295家。金融业不断壮大。昆仑银行、哈尔滨银行大庆分行正式挂牌，14家小额贷款公司开业，深圳创投、西部证券入驻我市。全市各项贷款余额410亿元、新增贷款86亿元。

　　（四）功能提升、管理创新，城市现代化建设迈出新步伐。实施重点工程60项，完成投资415亿元。集中建设功能项目。龙凤湿地跨线桥、公路客运枢纽、城市规划展示馆等12个项目竣工，铁路客运东西两站、哈大齐客运专线、中央党校大庆教学基地等项目加快推进，现代都市功能不断增强。整体开发建设新城。全面推进庆南、庆北新城建设，庆南新城林源工业区完成路网建设5公里，大同组团启动建设4.5平方公里新区，红岗组团完善教育、卫生等一批功能项目；庆北新城围绕兰德湖建设基础设施8项、完成投资2.6亿元。继续推进青龙山地区和龙凤湿地周边开发，拓展东部地区城市空间。富强村改造扎实推进，富强新区建设整体启动。全面加强现代管理。集中开展城乡环境综合整治，拆除违章建筑210万平方米，规范整治牌匾2.1万块，“门前五包”责任制进一步落实，网格化管理长效机制初步建立，“三城”创建成果巩固提升。持续搞好生态绿化。主干道沿路绿化230公顷，矿区环境绿化827公顷，城区重要节点景观绿化100处，人均公共绿地面积达到13平方米。系统完善规划设计。编制完善城市总体规划、城市空间发展战略和城市总体设计等5个基础性规划，以及交通、物流、湖泊治理等11个专项规划，规划水平整体提高。

　　（五）体制优化、活力增强，改革开放稳步推进。重点改革顺利进行。完成政府机构改革，推进国资监管体制改革，深化警务体制改革，实施文化体制改革，改革创新活力得到释放。对外贸易不断扩大。进出口总额完成15亿美元、增长36%，实际利用外资1.5亿美元、增长10%，中俄原油管道漠大线全面建成并正式输油。招商引资取得突破。引进项目526个，其中亿元以上项目62个、高新技术项目36个，到位资金188亿元、增长25.2%，沃尔沃、万达、美国爱科、挪威格兰等国内外名企入驻大庆，博润生物、大庆乳品厂成功上市，对外经济合作领域不断拓宽。

　　（六）民生改善、事业进步，社会建设不断加强。教育事业均衡发展。调整优化布局，撤并中小学校53所、改建新建22所，新增义务教育标准化学校79所，高考成绩保持全省领先。引进建设省旅游职业技术学院，高等教育不断提升。卫生事业持续发展。新建市妇女儿童医院、5所县区医院、8个社区卫生服务中心，龙南医院和市人民医院晋升三甲医院，城乡公共卫生服务体系进一步完善。文体事业蓬勃发展。市图书馆等重点功能项目顺利推进，新建乡镇文化站37个，完善农家书屋125个，“书香大庆”、“百湖之夜”、“十百千万”全民健身等群众文体活动深入开展。社保水平不断提高。“五险”净增16万人次，新农合参合率达到99.7%，7.5万企业退休人员养老金人均月增126元。社会管理创新完善。健全“四级”群众和社会工作网络，全面推行社区居站分设、选聘分开，顺利完成居委会换届，人口普查工作稳步推进。安全形势保持稳定。积极有效化解信访历史积案，深入开展打非治违专项行动和十大重点行业专项整治，安全生产事故下降15.1%，八类主要刑事案件下降20.2%，社会秩序和谐安定。

　　同时，十件民生具体实事全面落实。城乡新就业7.6万人，城镇登记失业率控制在4%以内；改造城市棚户区62.4万平方米，建设经济适用住房24.8万平方米，主城区棚户区改造基本完成；改造农村泥草房5万户，惠及农民17.5万人；城市生活垃圾无害化处理厂主体工程完工，市区10座垃圾中转站建成投用，四县城镇垃圾、污水处理设施基本建成；新增供热能力400万平方米，改造供热管网1.1万米，10万居民受益；东城水库竣工蓄水，东城水厂如期建设，改造供水管网1.5万米；道路交通控制中心及信息化交通控制系统建设如期推进；特殊教育发展中心开工建设；维修改造中小学校舍27.4万平方米；新增民办养老院10家、社区老年人日间照料室45个。　各位代表，过去一年取得的丰硕成果，为“十一五”划上了圆满句号。回顾过去五年的发展历程，我们在市委的坚强领导下，全力推进科学和谐跨越发展，经济转型和城市转型明显加快，可持续发展迈出重大步伐。

　　五年来，经济发展实现整体跨越。地区生产总值由1400亿元增长到2900亿元，地方经济增加值由310亿元增长到1100亿元，固定资产投资由302亿元增长到1036亿元，粮食总产由47.4亿斤增长到101.1亿斤，社会消费品零售总额由216.8亿元增长到591亿元，地方财政收入由70亿元增长到175.5亿元，主要指标都翻一番以上，实现历史性突破。以原油持续4000万吨稳产为标志，百年油田建设稳步推进；以120万吨乙烯改扩建为标志，石化产业战略升级；以沃尔沃汽车等产业龙头项目引进为标志，地方工业突破发展；以建成百亿斤粮食产能基地为标志，现代农业实现跨越；以北国之春梦幻城和新华08国际石油资讯中心开工建设为标志，服务业加快提升。地方经济总量突破千亿元，油与非油经济比例由65:35调整到52:48，万元gdp综合能耗下降20%，发展方式不断转变，经济结构持续优化。大庆综合实力位列全国地级城市第21位。

　　五年来，城乡面貌发生重大变化。城市框架全面拉开，启动建设庆南、庆北新城，加快东移北扩、南进西拓，城市规划区面积扩大188平方公里，建设重点工程171项，完成投资941亿元。立体交通体系全面构建，萨尔图机场通航，武警森林直升机场启用，铁路客运东西两站启动建设，大广高速大庆段全线贯通，中三路、萨大路等42项路桥工程竣工投用，城乡道路新增3738公里。生态环境整体优化，综合治理河湖17个，建设生态园89个，建成区绿化覆盖率由35.6%提高到45%，农村植树造林80万亩，全市森林覆被率由8.5%提高到10.6%，城镇生活垃圾无害化处理率由68%提高到85%，污水处理率由51%提高到90%。城市管理水平明显提高，环境综合整治持续开展，主城区数字监控基本覆盖，城市管理体制和长效机制更加完善。大庆成为全国集国家环保模范城、国家卫生城和全国文明城“三城”品牌于一身的七个地级城市之一。

　　五年来，人民生活水平连上台阶。居民收入大幅增加，城镇居民人均可支配收入由13662元提高到20016元，农民人均纯收入由2723元提高到8045元。生活条件大幅改善，农村自来水普及率由43%提高到72%，城市天然气入户率由20%提高到80%，城镇人均居住面积由32.9平方米提高到39.3平方米，农村住房砖瓦化率由36.6%提高到72.3%。保障水平大幅提高，新增就业21.8万人，“五险”净增98万人次，在全省率先实行全民医保，在全国率先建立城市“十位一体”和农村“六位一体”低保综合救助体系，市区最低工资和城市低保标准保持全省最高。大庆成为全国42个相对富裕城市之一。

　　五年来，社会事业得到大幅提升。教育事业更加均衡优质，义务教育标准化学校达到172所，省级示范高中发展到13所，国家和省重点中职学校发展到10所，高等院校发展到8所。医疗卫生体系更加健全，新建市级和县区级医院9家、乡镇卫生院51所、社区卫生服务机构165个，三甲医院发展到5家，社区和村级卫生服务实现基本覆盖。文体事业更加繁荣，铁人纪念馆、石油科技馆、歌剧院、博物馆、青少年科技活动中心等一批重要场馆建成使用，奥林匹克公园和西城体育馆等开工建设，文化创意产业园加快推进，三届湿地旅游文化节成功举办，特色文化精品不断涌现，群众文体活动蓬勃开展。大庆公共文明指数位列全国地级城市第19位。五年来，改革开放迈出重要步伐。深化政府机构改革，基本完成驻外办事处改革，推进审批制度改革，行政管理体制更加顺畅；扁平管理，下沉警力，警务体制改革走在全国前列；国库集中支付，建立“取消报名、低价中标、差额履约”的政府投资项目招投标体制，财政资金使用效率大幅提升；新闻传媒和文艺院团改制，实行集团经营，文化产业实现新发展。积极开展跨区域交流合作，与104个境外城市和地区建立经贸合作关系，与国外7个城市结为友好城市，招商引资累计到位资金674亿元，外贸进出口总额增长4.6倍。引导鼓励全民创业，非公企业由7600家增长到13800家，非公经济增加值占gdp比重由14.7%提高到19.9%、占地方经济比重达到60%，上市企业由4家发展到17家、累计融资近百亿元。

　　五年来，社会始终保持和谐稳定。构建大信访、大调解、大防控、大安全工作格局，建立“四级”群众和社会工作网络，实行“六调联动”工作机制，成立信访调处中心，推行重大事项社会稳定风险评估、领导干部接访包案、信访稳定督查等制度，社会不稳定因素得到及时化解，社会管理体系不断完善。八类主要刑事案件下降61.2%，群众安全感明显增强。生产事故下降68.2%，安全生产形势持续稳定。建立城市公共安全应急指挥中心，妥善处理自然灾害和突发公共事件，应急处置能力切实加强。圆满完成奥运火炬传递、国家领导人视察等重大安保任务，社会安全指数达到96%以上，“五个确保”目标基本实现，平安大庆建设成效显著。

　　各位代表，“十一五”取得的发展成就，得益于市委统揽全局、坚强领导；得益于人大和政协有力监督、大力支持；得益于各民主党派、工商联、无党派人士和群团组织建言献策、积极参与；得益于中直石油石化大企业及驻庆中省直单位鼎力相助、携手共建；得益于全市上下团结奋斗、不懈努力。在此，我代表市政府向全市人民和所有为大庆发展做出贡献的同志们、朋友们，表示崇高的敬意和衷心的感谢！

　　总结成绩的同时，我们也必须清醒地看到存在的现实问题。主要是经济结构不够合理，接续产业层次有待提升，立市立县立区的大项目、战略新兴产业项目需要加快建设，发展方式亟待转变；承载发展的市政基础不够坚实，城市功能需要持续完善；城乡二元结构矛盾较为突出，农村生产生活条件需要加快改善；公共服务体系不够健全，社会管理存在薄弱环节，群众的一些新期待新诉求需要更好满足；个别公务人员服务意识不强、工作效率不高，作风需要进一步改进，政府效能建设有待加强。二、“十二五”发展目标和2011年主要工作

　　“十二五”是大庆打牢发展基础的全面推进期，是建设全面小康社会的攻坚决胜期。我们必须认清形势，明确责任，忠诚使命，开拓进取，努力构筑新优势、创造新生活。

　　新的阶段，我们要倍加珍惜机遇潜力。国家坚持实施扩大内需战略，支持东北地区等老工业基地振兴，促进资源型城市转型，可利用争取的政策环境更好；省里全力推进“八大经济区”、“十大工程”，扩张升级“十大产业”，可承接拓展的产业空间更大；中油集团加快建设“五大基地”，支持石化延伸增值，保障地方企业原料需求，可吃配加工的下游领域更宽；原油继续保持4000万吨稳产，中俄原油管道全线输油，地企形成强大合力，可依托的发展根基更实；120万吨乙烯改扩建、30万吨聚丙烯二期、沃尔沃乘用车生产基地等一批龙头项目将建成投产，可催生释放的内在动力更强。这都需要我们牢牢把握战略机遇，深挖多方潜力，发挥综合优势，不断夯实发展基础。

　　新的阶段，我们要勇于面对压力挑战。经济发展既要立足翻番确保增长速度“快”，更要突出科学发展主题和转变发展方式主线，确保质量效益“好”、内部结构“优”，是一个务必突破的重大课题；既要集中完善都市功能，加强现代城市管理，有效防治“城市通病”，更要推进城乡一体化，破解二元结构矛盾，建设全面小康社会，是一个务必完成的重大任务；社会结构和利益格局深刻调整，社会主体多元性和选择性增强，既要提升优化公共服务层次，更要增强可及性和公平性，维护社会和谐稳定，是一个务必直面的重大考验。这都需要我们传承发扬大庆精神、铁人精神，变压力为动力，攻坚克难，勇于突破，不断创造新的业绩。

　　新的阶段，我们要主动承担责任使命。胡锦涛总书记、温家宝总理指示大庆“推动科学发展”、“实现城市转型”，吉炳轩书记、王宪魁省长要求大庆“更好更快发展”、“争当全省排头兵”。特别是市委七届九次全会明确提出了“提前五年建成全面小康社会，让发展基础更坚实，让人民生活更幸福”的奋斗目标。这一奋斗目标，是贯彻落实中央和省委殷切希望的实际行动，是实现可持续发展的必然选择，是新时期赋予大庆人的责任使命。实现这一目标，就能确保大庆发展走在全国前列，确保大庆发展内生动力不断增强，确保全市人民共享发展成果。这都需要我们负责任地干事、干负责任的事，创新创造创优，大干快干实干，不辱发展使命，不负党和人民重托。

　　“十二五”期间，我们要认真贯彻落实市委七届九次全会精神，坚持以科学发展为主题，以转变经济发展方式为主线，围绕“提前五年建成全面小康社会，让发展基础更坚实，让人民生活更幸福”的奋斗目标，全面实施“一二三四五”发展战略，大力调整产业结构促进经济快速健康发展，整体改善民生全面加强社会建设，统筹推动城乡发展加快城市化和城市现代化，不断创新体制机制深化改革扩大开放，全力加快大庆科学和谐跨越发展。按照市委七届九次全会提出的要求，未来五年重点完成五大战略任务：　——创建百年油田。坚持“发展共谋、责任共担、城乡共建、稳定共抓”方针，支持油田“增资源、保稳产、转方式、夯基础、提效益”，到2015年，实现原油高效益、可持续、有保障的4000万吨稳产。支持天然气勘探开发，力争油气当量稳中有升。支持多种经营、多业突破，未上市业务经营收入达到800亿元。支持开发国际市场，实现海外业务跨越发展，经营收入达到400亿元。支持和谐示范矿区建设，一年见成效、三年大改观、五年基本建成，百年油田建设再创新辉煌。

　　——壮大接续产业。围绕打造“5个销售收入超千亿的集群产业和10个销售收入超百亿的支柱产业”战略，推动石油化工、商贸流通两个销售收入已超千亿元产业实现倍增，分别达到2000亿元以上；做大油化装备、新兴装备，推动现代装备产业销售收入超千亿；突破发展汽车产业，加快形成整车生产和关联配套能力，实现汽车制造业销售收入超千亿；农产品深加工、新材料、新能源、建筑安装、旅游、电子信息、服务外包、生物工程、文化创意、现代物流10个产业销售收入分别实现超百亿。三次产业结构更加优化，绿色低碳理念充分体现，现代产业体系初步建立。2015年地区生产总值和地方财政收入分别比2010年翻一番，非油经济比重达到60%，基本实现经济转型。

　　——打造宜居城市。坚持“生态、自然、现代、宜居”的城市建设理念，加快城市现代化步伐，基本实现城市转型，建成现代化国际化大城市。全面拉开城市框架，构建主城区核心组群、四县卫星组团、中心镇特色组团梯次衔接的现代城市布局体系。推进新城开发旧城改造，老区提升功能补位，集中建设配套完备的现代城市功能体系。增加航线航点开通国际航班，建成东西城铁路枢纽，开通城际列车，升级高速公路，畅通市区交通，完善农村路网，建成外通内畅的现代城市交通体系。实施植树、复草、治水、净气、降噪工程，建设防护林带，恢复草原植被，建成区绿化覆盖率达到45.5%，打造健康完整的现代城市生态体系。巩固“三城”创建成果，健全“四级”城管体制，落实“五化”机制，建立高效运行的现代城市管理体系。

　　——建设社会主义新农村。着眼实现农村全面小康，加快推进城乡一体化，整体提升农村现代化水平。发展现代农业，巩固提升百亿斤粮食产能，畜牧业占农业比重达到60%，棚室规模达到30万栋以上，建成东北地区最大的蔬菜生产供应基地。建设现代农村，改造升级乡村道路1500公里，基本建成新型农村电网，全面普及自来水、有线电视、互联网，城关镇垃圾集中收集处理率达到80%以上，绿化村屯800个，完成造林70万亩。培养现代农民，提高文化、技术、健康、文明等综合素质，增强创业创收和经营管理能力，更好地发挥社会主义新农村建设的主体作用。

　　——构建和谐大庆。满足基本民生，建设小康民生，发展现代民生，城镇居民人均可支配收入年均增长12%以上、达到35500元，农民人均纯收入年均增长15%以上、达到16000元。优先发展教育事业，提前五年在全省率先基本实现教育现代化。提高医疗卫生水平，建立起覆盖城乡、优质便民的医疗服务体系。积极发展文化事业和文化产业，打造中国北方文化名城。创建国家级创业型城市，城镇登记失业率控制在4.5%以内。推进城乡医保、城乡低保、城乡养老保险统筹发展，形成大保障格局。健全社会管理组织，创新社会管理方式，增强公共安全应急处置能力，妥善化解各类社会矛盾，打造平安大庆。各位代表，2011年是“十二五”起始之年，开好局起好步至关重要。全市经济社会主要预期指标是：地区生产总值增长12%以上，全社会固定资产投资增长35%，地方财政收入增长20%以上，城镇居民人均可支配收入、农民人均纯收入分别增长12%和15%。

　　（一）服务油化经济，夯实持续发展基础。围绕“五大基地”建设，全力以赴为油化大企业搞好生产生活服务。支持油田4000万吨产能建设。大力整治生产环境，拆除占压油气管线建筑，加快还油还绿，共建绿色油田。严厉打击破坏产能设施等违法犯罪活动，维护正常生产秩序。全力保障油田用地、勘探开发等发展需要，服务原油持续高效稳产。推动石化产业延伸增值。支持中直石化大企业扩能上项目，服务120万吨乙烯改扩建、千万吨炼油、30万吨聚丙烯二期等石化大项目加快建设。地企联合谋划建项目，着力推进50万吨c4综合利用、36万吨eps等重点项目，促进华科、飞马、联塑等地方企业与石化大企业协作，搞好吃配延伸。对外招商引项目，围绕聚烯烃、合成橡胶、液态化工、炼油副产、塑料助剂等重点领域，大力引进战略投资者，推进下游精深加工。石化工业增加值增长15%以上。

　　（二）坚持项目牵动，加快壮大地方工业。突出抓项目抓企业抓品牌，地方工业增加值增长40%以上。全力推进项目攻坚。落实省里打好产业项目“三年攻坚战”部署，全市新建续建投资千万元以上地方工业项目256个，其中亿元以上项目74个，确保建成投产197个。突出抓好沃尔沃乘用车生产基地、忠旺铝材等龙头大项目，着力推进大丰抽油机、爱科和格兰农机具、伊利婴幼儿奶粉、国电风力发电等重点项目，支撑地方工业加快发展。扶持企业做大做强。支持中蓝石化、展华生化等企业扩产能、拓市场，扶持路通、井泰等企业挂牌上市，新增主营业务收入超亿元企业15家、超5亿元企业4家、超10亿元企业2家，新创省级以上名优品牌18个，增强企业市场竞争力和品牌影响力。推动园区承载升级。坚持资源共享、政策一致、利益互惠，鼓励县区发展“飞地”经济，举全市之力推进园区经济规模发展。高新区以建设战略新兴产业城为牵动，突出引建大高外新项目，加快国家创新型科技园区建设，工业增加值增长45%以上。经开区以建设国家级经济技术开发区为目标，集约发展装备制造和新能源汽车等产业，工业增加值实现倍增。林源工业区以启动建设550万吨重油催化热裂解项目为重点，着力做好石化延伸吃配，加速壮大园区规模实力。增强人才科技支撑。围绕重点产业发展和项目建设，建立专家工作站、工程技术中心，突出引进领军人才和研发团队。推行“政府引导+企业投资+风险投资+科研院所”模式，完善孵化器、科技成果交易中心等载体功能，重点对接转化生物、新能源、新材料等领域技术成果，助推产业升级发展。

　　（三）推进深度开发，繁荣发展服务业。适应多样化消费需求，打造都市休闲经济，做活生活服务业；适应经济快速扩张和发展方式转变，做大生产服务业。服务业增加值增长20%以上。多层次发展商贸业。加快建设东城万达广场和西城万达中央商务区，引进建设红星美凯龙、松雷等名企名店，打造高端商贸；扩大城市社区连锁，拓展农村便民连锁，新增社区店60个、农家店300个，完善利民商业网络；组合开发商旅文综合街区，丰富提升新型业态。分类别突破旅游业。突出石油文化、湿地、温泉、草原风情等特色旅游，努力打造外来人“留得住”、本地人“游起来”的旅游目的地。建好龙凤国家城市湿地公园，提升当奈湿地、鹤鸣湖、莲花湖景区，把湿地旅游做大；以杜尔伯特连环湖、林甸北国温泉养生广场为牵动，围绕“休养保养疗养”打造温泉文化，做大做精温泉旅游产业；依托铁人纪念馆、油田历史陈列馆等场馆，系统策划开发红色旅游。加快北国之春梦幻城等重点旅游项目建设，扎实做好旅游线路、旅游产品、旅游品牌的整体设计和包装推介，旅游收入增长30%以上。抓创意引领文化业。着眼满足市民日益增长的物质文化需求，创意开发休闲文化产品，加快建设百湖影视基地、黑鱼湖国际艺术村等项目，提升文化创意产业园规模层次。抓好新华08国际石油资讯中心等项目，带动发展石油资讯、信息服务等产业。培育现代传媒、演艺娱乐、文博会展、体育赛事等文化产业市场。聚要素做活物流业。服务石化生产，建好卓洋、神舟等石化产品物流园区。扩大粮食流通，建好新站、太阳升等粮食物流园区。发展棚室经济，建好林甸四季青、大同八井子等果蔬物流园区。建体系培育金融业。出台支持金融业发展政策，充分发挥现有金融机构作用，引进浦发、招商、兴业等银行机构，新建10家小额贷款公司，组建创业投资公司和融资性担保公司，鼓励发展证券、保险、风险投资，构建多元金融体系。支持创新金融产品，深化银企对接，促进金融与地方经济互动发展。壮规模扩张外包业。建设服务外包产业园三期，推进黑龙江软件园，引进大唐通信呼叫中心运营基地、世纪互联云计算中心。建设5万平方米数据中心和高新区物联网基础工程，努力打造世界级石油石化服务外包基地。服务外包企业发展到400家。　（四）统筹实施“双十”工程，提升新农村建设水平。推动“四大基地”集约高效发展，加快小城镇和中心村建设，突出壮大棚室经济，夯实农业农村发展基础。农业增加值增长12%以上。抓好农村民生“十项工程”。提升农村功能环境，新建饮水安全工程230处、大型沼气工程2处，实施农村电网升级改造工程11项，建设农村公路228公里，整体绿化村屯200个。完善农村公共服务体系，撤销村级小学24所，新建和改扩建农村寄宿学校11所；改造建设乡镇卫生院3个，完善村卫生所162个；建设改造乡镇文化站17个，新增有线电视入户3.5万户、上网村30个。推进农村基础建设“十项工程”。巩固百亿斤粮食产能，新建膜下滴灌156万亩、旱田喷灌53万亩，整理改良土地8850亩，高产粮食基地达到650万亩。提高畜牧业标准化水平，推进奶牛养殖“三进三退”，新建改建奶牛小区和牧场100个。做精做优粮食品牌，绿色食品基地面积达到500万亩，销售收入增长10%以上。提高农业生产组织化程度，新组建现代农业农机专业合作社20个，农村合作经济组织达到540个。扩张发展棚室经济。建好四季青温泉果蔬、大同棚室水果、连环湖旅游观光采摘等7个特色基地，八井子乡、福兴乡、杏树岗镇、春雷农场等60个千亩特色果蔬园区，新建棚室10万栋。推进小城镇建设。系统完善规划，加速城关镇提档升级，逐步推进近郊镇撤镇建街，有序抓好撤屯并村，优化小城镇建设布局。以连环湖、四季青、朝阳沟、新站、高台子等10个示范镇为牵动，加快配套基础设施和公共服务，突出培育壮大特色产业，努力打造一批工业强镇、商贸大镇、旅游名镇。

　　（五）加大建设力度，提升城市功能形象。推进68项城建重点工程，加快城市化和城市现代化，推动生态自然现代宜居城市建设取得新突破。重点开发建设城市新区。在加快庆南、庆北新城建设的同时，开发15平方公里的庆东新城和38平方公里的庆西新城。重点开发10个区块：沿龙凤湿地周边启动建设庆东新区，结合铁路大庆东站建设扩展完善青龙山地区，启动侏罗纪公园加快建设北国之春梦幻城，围绕兰德湖开发新的居住社区，在铁路大庆西站站前建设新的中央商务区，结合富强村改造开发建设富强新城，着眼改善油田职工居住条件高标准建好乘风新区，继续推进红岗、大同、林源三个组团加快庆南新城市政建设，结合让胡路商厦地区改造建设新的商业中心，结合高新区旧城改造建设现代新型功能社区。高标准建好重点项目。加快建设奥林匹克公园、图书馆、妇女儿童医院、西城区青少年活动中心等重点功能项目，不断满足市民生活需求。全力推进东城水厂、城市生活垃圾无害化处理厂、垃圾中转站、矿区生活污水截流、油田热电厂扩建、供水管网改造等市政设施建设，提高城市承载能力。加快改造南一路、北一路，建设南外环路，新增东西两城3条快速通道；打通经二街、经六街、经七街等6条瓶颈路，延伸乘风东路、龙十路、龙化路等5条断头路，升级改造外环东路、奥林路、中八路、创业大道，建设k168、k145两座立交桥，提升城区通行效率。加大城市整理力度。清理违章建筑，拆除主要街路两侧破旧厂区和低档商服，实施“线杆下地”，规范户外牌匾，更新完善亮化设施。加快主城区“退二进三”，对不符合空间布局的工业区实行规划调整。持续抓好生态建设。新建城市园林30处，实施滨洲湖等8个湖泊环境改造工程，绿化大广高速大庆段、西一路等22条干道，建好城区周围片状林，新建城市绿地1500公顷、植树200万株，人均公共绿地面积达到13.5平方米。强化现代城市管理。继续开展十项城乡环境综合整治，严格落实“门前五包”责任，深入推进网格化管理，更新保洁、清雪、养护、维修等现代装备，扩大数字城管覆盖范围，逐步实现城市全域精细管理，确保“三城”创建成果持续巩固提升，顺利通过国家环保模范城市复检。

　　（六）切实改善民生，着力加强社会建设。围绕构建“五和”社会，满足基本民生、建设小康民生、发展现代民生，进一步提高人民群众幸福指数。办好群众关心的具体实事。一是建设幼儿园，规范提升400所民办园，新建改建城乡140所公办园。二是改善公共交通，优化新增公交线路8条，更新车辆150台，增加驾驶员120名。加强出租车市场管理，整顿运营秩序。三是建设停车场，新建停车场24个，增加停车泊位1万个。四是解决农民看病问题，培训乡村医生900人，按政策提高农民大病救助标准，农村基层卫生机构设施设备完善率提高35%。五是促进大学生就业，落实好国家政策，用好市人才发展专项资金，创造就业岗位5000个。六是改善低收入群体住房条件，建设公共租赁房4万平方米，结合棚户区改造建设经济适用住房15万平方米，改造农村泥草房3万户。七是改造老居住区，全面加快东风新村、万宝小区、景园小区等28个老居住区改造，市区争取年内全部完成。八是完善社会化养老服务，建设民办养老院13个、社区老年人日间照料室90个。九是加强物业行业管理，规范业主委员会选举程序，完善物业企业服务标准，建立物业企业辞退机制。十是完善社区文化休闲功能，新建20个社区文化活动中心、100个健身场地。提高公共服务水平。加快发展教育事业，撤并中小学校27所，维修改造中小学校80所，新增义务教育标准化学校40所，扩大优质高中教育覆盖面，启动建设中等职业教育园区和大型公共实习实训基地，加强高校与地方深度融合。

　　全面发展卫生事业，整体提升综合医院医疗水平，建好眼病、糖尿病等八大特色专科，新建社区卫生服务机构35个，在全市基层公立医疗卫生机构全面推行基本药物制度。繁荣发展文体事业，加快推进博物馆二期建设，推出电影《王进喜》等艺术精品，打造60个特色文化社区和文化大院，深入实施“十百千万”工程，促进全民健身活动广泛开展。完善城乡社会保障体系，“五险一金”扩面净增4万人次以上，按政策提高退休职工基本养老金和最低工资标准，鼓励创业促进就业，新就业5万人，城镇登记失业率控制在4.5%以内。营造和谐安定的社会环境。按照“居站分设、选聘分开”模式，推进社区社会工作站全覆盖。完成第九届村委会换届选举，选好新农村建设带头人。加强社会治安综合治理，严厉打击各类违法犯罪，维护良好的经济和社会秩序。突出抓好食品、药品、交通、建筑、生产、消防、校园、矿区等八个重点领域安全监管，提高快速应急反应和救援能力。重点防治和妥善处理征地拆迁、劳资矛盾、土地纠纷等问题，保持社会和谐稳定。 　（七）深化改革开放，增强发展活力。积极推进重点改革。不断深化财政体制、文化体制、投融资体制、警务体制等项改革，全面推进医药卫生体制改革，继续完善农村综合改革，深入探索要素市场改革。全面拓展对外合作。招商引资重点在战略龙头项目上突破，争取引进超亿元项目50个以上、到位资金150亿元。对接争取国家扩大内需的有关政策、省里发展“十大产业”的项目支持，与石油石化大企业携手向中油集团争取石化延伸增值项目，积极扩大与省直厅局及友好城市的交往合作，进一步营造有利于更好更快发展的外部环境。组织漠大线管道原油进口贸易，积极争取1500万吨俄油贸易额记在大庆并做销。重点支持外向型生产企业发展，新增有进出口业绩企业30家，进出口贸易总额增长20%以上。加快发展非公经济。落实民营经济发展政策，完善创业担保体系，搞好创业培训，加快建立现代企业制度，力争非公经济增加值增长25%以上。

　　（八）改进作风提升效能，加强政府自身建设。认真贯彻落实市委《关于加强作风建设提高行政效能的决定》，深入开展作风建设年活动，继续推进行政提速、管理创新、执行彻底、结果问效，增强政府执行力，确保高效完成各项目标任务。按照“破满、破难、破旧、破靠”的要求，解决好思想问题。坚决克服满足现状，倡导争创一流，形成比学赶超的生动局面；坚持把思维起点放在干事成事上，困难面前不说不行，千方百计想办法怎么能行；切实摒弃惯性思维和陈旧观念，善于创新举措解决问题；自觉主动想事谋事，做到定下的事快干，安排的事快办。克服“十弊”，解决好作风问题。对照“十弊”现象，深入查摆存在问题，认真剖析原因症结，力求早发现、早防范。针对存在的突出问题，研究制定方案，明确整改措施，保证问题整改到位。加强制度建设，努力从源头上、根本上防止和克服“十弊”现象发生。立足“两个提高”，解决好工作机制问题。坚持“三维分工”、“五定五保”、重点项目指挥部制，完善定期通报、现场办公、“三位一体”督办，推行并联审批、网上审批和审批服务代办制度，创新产业项目推进、经济综合考评等机制办法，确保执行落实的力度和推动发展的效能。

　　始终坚持市委统一领导，主动接受人大法律和工作监督，自觉接受政协民主监督。认真贯彻落实市委决策，认真执行人大及其常委会决议，认真办理人大代表建议和政协委员提案，广泛听取民主党派意见建议，支持工会、共青团、妇联等群团组织开展工作。坚持重大问题集体讨论决定，健全社情民意反映机制和重大事项专家咨询、社会公示制度，确保科学民主决策。推进依法行政，规范行政许可行为，努力提高政府的法制化管理水平。

　　各位代表，“十二五”是大庆承前启后、继往开来的重要时期，加快经济社会发展责任重大、使命光荣。让我们在市委的坚强领导下，紧紧团结依靠全市各族人民，继续传承和弘扬大庆精神、铁人精神，解放思想、与时俱进，居安思危、团结拼搏，为实现“十二五”良好开局、建设全面小康社会而努力奋斗！
